# A Thematic Inquiry into the Burnout Experience of Australian Solo-Practicing Clinical Psychologists

**DOI:** 10.3389/fpsyg.2017.01996

**Published:** 2018-01-19

**Authors:** Trent E. Hammond, Andrew Crowther, Sally Drummond

**Affiliations:** ^1^Division of Student Services, Charles Sturt University, Sydney, NSW, Australia; ^2^School of Nursing, Midwifery and Indigenous Health, Faculty of Science, Charles Sturt University, Albury-Wodonga, NSW, Australia; ^3^School of Nursing, Midwifery and Indigenous Health, Faculty of Science, Charles Sturt University, Wagga Wagga, NSW, Australia

**Keywords:** burnout, depersonalization, emotional exhaustion, lived experience, personal accomplishment, semi-structured interview, thematic analysis

## Abstract

**Objective:** Burnout is conceptualized as a syndrome that consists of emotional exhaustion, depersonalization, and decreased personal accomplishment. Despite the increased frequency and severity of burnout in the Western world, there is limited published research regarding the experiences of clinical psychologists who have had burnout. The present study examines clinical psychologists’ different experiences of burnout in Australia.

**Design and Methods:** In the year 2015, six privately practicing and solo-employed clinical psychologists provided rich qualitative data by participating in semi-structured interviews. Thematic analysis was the method used to analyze clinical psychologists’ natural accounts of their burnout experiences. Using NVivo, emerging themes were identified through coding ‘first order constructs’ and then axial code ‘second order constructs.’

**Findings:** Clinical psychologists indicated that their roles are demanding and a diverse range of symptoms, including the enduring effects of burnout, mental stress, fatigue, decreased personal accomplishment, negative affect, depersonalization, reduced productivity and motivation, and insomnia. They identified precursors of burnout, including excessive workload and hours of work, life stresses, mismanaged workload, and transference. Clinical psychologists suggested that protective factors of burnout include knowledge and years worked in direct care, and trusting and long-term relationships. They indicated that the barriers to overcoming burnout include the fallacy that their clients’ expectations and needs are more important than their own, the financial cost of working in private practice, contemporary knowledge and inadequate education regarding self-care, and time constraints.

**Discussion and Conclusion:** The findings presented in this study provide psychologists and other health professionals with an insight about the burnout experience and inform professionals of the mental shortcomings of working as a solo-practicing clinical psychologist. Findings from this study should lead to an increased understanding of the complexities of burnout, and ultimately reduced cases of burnout, absenteeism, and staff disengagement.

## Introduction

The term ‘burnout’ was originally suggested by Freudenberger (1974), who observed substance-abuse users who stared at cigarettes until they burnt-out. Occupational burnout is conceptualized as a syndrome that results from overwhelming work-related mental stress and is considered to consist of three domains, namely, emotional exhaustion, depersonalization, and decreased personal accomplishment (Maslach and Jackson, 1981a). Emotional exhaustion is defined as the complete depletion of one’s emotional resources. Depersonalization refers to cynical, negative, callous, or dehumanizing perceptions of people who are usually clients. Reduced personal accomplishment occurs when one perceives one’s self and achievements negatively. Although burnout is not a disorder, according to the Diagnostic and Statistical Manual of Mental Disorders (American Psychiatric Association, 2013), it is a “life management” problem, as per the International Statistical Classification of Diseases and Related Health Problems (World Health Organization, 2015).

Maslach’s (1982) burnout model suggests that interpersonal demands, or ‘mental stress,’ result in increased emotional exhaustion, heightened depersonalization, and decreased personal accomplishment. Emotional exhaustion and the lack of concern for clients indirectly results from distancing one’s self from one’s clients. Depersonalization is theorized to stem from emotional exhaustion, and it is suggested that reduced personal accomplishment stems from depersonalization. The opposite of burnout is engagement, which is characterized by energy, efficacy, and involvement (Maslach and Leiter, 1997).

Maslach and Leiter (1997) identify six risk factors for burnout: (1) Lack of control – people who are burnt-out do not feel content with their careers and have limited autonomy in their work roles. (2) Insufficient reward – people who lack satisfaction at work (for example, due to lack of pay, insufficient benefits, lack of recognition, and/or limited intrinsic reward) often experience burnout. (3) Lack of community – people who are burnt-out tend to have less positive connections with their colleagues, difficulties working in teams, and problems with their professional relationships. (4) Perceived lack of fairness – people who are burnt-out may believe that they have been disrespected or may feel powerless. Inequitable pay structures, cheating at work, inappropriate promotions, and poor dispute resolution practices can lead to a sense that one’s workplace is unjust and unfair. (5) Conflict in values – people whose values and goals do not align with their organizations’ vision and mission are susceptible to burnout. (6) Work overload – burnt-out people often have an unsustainable workload, limited time available, and perform overly difficult jobs.

The Maslach Burnout Inventory (MBI) was constructed based on exploratory research that consisted of a variety of 1,025 human service professionals (Maslach and Jackson, 1981a). Researchers analyzed the data using principal factor component analysis of nurses, medical practitioners, health assistants, social workers, counselors, psychotherapists, police officers, correctional officers, and clergy staff. Since Maslach and Jackson (1981a) published their seminal MBI, thousands of researchers have investigated the topic of burnout. For example, an advanced search using ProQuest reveals that between 1981 and December 22, 2017, 9,204 scholarly journals were published containing the document title keyword ‘burnout.’ Research regarding burnout has been predominantly quantitative (Ackerley et al., 1988; Huberty and Huebner, 1988; Raquepaw and Miller, 1989; Huebner, 1992, 1993, 1994; Sandoval, 1993; Rupert and Morgan, 2005; Lee et al., 2011). Quantitative research mainly focuses on identifying the key characteristics of burnout by using the MBI as a measure. There is currently no published qualitative research in English that provides information about clinical psychologists’ experiences and how they cope with burnout, as conceptualized by Maslach and Jackson (1981a).

However, a recent article reports on the experiences of 28 substance abuse treatment counselors who participated in focus groups in Kentucky, United States of America (United States) (Oser et al., 2013). The authors used Maslach and Jackson’s (1981a, p. 19) construct of burnout to answer the question “Do you or counselors you know ever feel “burned out?”. The semi-structured interviews provided counselors with opportunities to discuss a variety of topics. The counselors discussed the factors impacting client behavioral treatment outcomes from their own perspectives. Topics included counselor characteristics, burnout, views of pharmacological treatment, and other less invasive treatment options. Oser et al. (2013) identified, by following grounded theory methodology, that counselors believe that burnout is caused by challenging and difficult clients, large caseloads, excessive paperwork, office politics, and low prestige. Further, burnout may be prevented by co-worker support, clinical supervision, and self-care. The consequences of burnout include poor client care, reversing roles, clients changing counselors, and job turnover.

Lee et al.’s (2011) meta-analysis of published research during 1988–2008 provides informative but correlational findings of psychotherapist burnout in the United States. Lee et al. (2011) analyzed the data of a variety of studies that recruited psychotherapists, including counselors, licensed psychologists, and clinical psychologists. By analyzing Pearson correlation coefficients, those researchers found moderate effect sizes for psychotherapists’ over involvement and control of their work. Like Rupert and Morgan (2005), emotional exhaustion was reported to have a positive correlation with over-involvement. Personal accomplishment was directly related to increased control. Lee et al. also identify that emotional exhaustion is related to increased job turnover intention and decreased job satisfaction. Depersonalization also had a positive correlation with decreased job satisfaction.

Farber and Heifetz’s (1982) article describes published qualitative research involving 60 health professionals. Those professionals were based in the United States and included private and public practicing psychologists, psychiatrists, and social workers. Through summative content analyses of interview transcripts, Farber and Heifetz identified that more than half of those professionals had burnout. They described problems relating to their clients, including ‘non-reciprocated’ attentiveness, constantly ‘giving’ one’s self to clients, and increased personal responsibility for clients. Three-quarters of the health professionals believed that unsuccessful client outcomes resulted in heightened stress. About one-fifth of the health professionals suggested that overworking resulted in their burnout. These results align with those of Rupert and Morgan (2005), who found that solo practicing psychologists experience over-involvement and constantly feel responsible for their clients.

Burnout has negative implications for clinical psychologists and other professionals employed both within and outside of the health and community services industry. Triggers of burnout include work pressure, heavy caseloads, occupational violence, trauma, attempted suicide, harassment, and bullying (Raquepaw and Miller, 1989; Safe Work Australia, 2013a). Clinical psychologists regularly deal with complex cases and treat clients who have severe mental illnesses and show little improvement (Acker, 2012). Long-term client support, management of personal priorities, advocacy, and stringent referral processes increase the burden placed upon clinical psychologists (Pines, 1993; Scheid, 2003). Psychologists are susceptible to burnout because of excessive demands, pressure to provide efficient and cost-effective services, community expectations, and ongoing changes in health policies (Health Workforce Australia, 2014). Australian statistics for burnout are unavailable for health professionals, but international statistics show that burnout of clinical psychologists ranges between 8 and 40 percent (Ackerley et al., 1988; Huebner, 1992; Sandoval, 1993).

There are several consequences of becoming burnt-out. Severe burnout leads to headaches, nausea, suicide, chronic fatigue, ulcers, gastric-intestinal disorders, frequent and prolonged sickness, and hypertension (Schaufeli and Enzmann, 1998). In Australia during 2003–2013, mental stress was associated with 95 percent of mental disorder workers’ compensation claims (Safe Work Australia, 2013b). The median cost of mental stress claims to Australian employers was over ten billion dollars. The average lost work time due to mental stress was 5 weeks. The greatest proportion of claims, 21 percent, were from health and community services industry employees. That industry consists of over 22,000 psychologists (Safe Work Australia, 2013b; Health Workforce Australia, 2014), and approximately 29 percent are endorsed in clinical psychology (Psychology Board of Australia, 2014).

The open-ended research question explored in this study was “what are clinical psychologists’ experiences of burnout?” The question provided a framework to understand the experiences of Australian clinical psychologists with burnout and to analyze interviews and compare emerging themes. The participants’ experiences of working as clinical psychologists in solo private practice were illuminated, including the implications of burnout, and the factors that led to their burnout.

## Materials and Methods

### Study Design

The intention of the study was to allow for the emergence of Australian clinical psychologists’ experiences of burnout during individual in-depth interviews. Theoretical essentialist thematic analysis (Braun and Clarke, 2006) was the research methodology selected to form a detailed account of clinical psychologists’ burnout experiences. The method is flexible and is not wedded to any pre-existing theoretical framework and allowed the researchers to gain insight into the situations leading up to burnout and the experiences of clinical psychologists.

Berger and Luckmann’s (1966/1991) “social constructivist” worldview somewhat influenced the way that the raw data were collected and analyzed. The underpinning assumption of the study was that clinical psychologists would be willing to share their experiences of burnout and openly identify that they had experienced the syndrome. Clinical psychologists answered a series of open-ended questions to allow for inductive exploration. The participants’ reactions, behaviors, thoughts, impressions, feelings, interpretations, and understandings became apparent in the data. At the time that clinical psychologists were interviewed, the researchers believed that their experiences were shaped and constructed through both their subjective experiences and the experiences of others.

The study was carried out in accordance with the recommendations of Charles Sturt University’s Human Research Ethics Committee (Protocol Number: 2014/223) with written informed consent from all participants. The participants gave their informed consent in accordance with the Declaration of Helsinki (World Medical Association, 2013) and Australia’s National Statement on the Ethical Conduct of Human Research (National Health and Medical Research Council, 2007). Participant data will be retained for up to 5 years and then deleted in the year 2021.

### Participants

Clinical psychologists’ contact details were obtained, in line with the methodology of purposive (criterion-based) sampling (Patton, 2002), from both the Australian Psychological Society’s (2017) and the Australian Clinical Psychology Association’s (2017) online membership directories. Their names were screened against the National Register of the Australian Health Practitioner Regulation Agency (AHPRA) before being contacted by email. AHPRA regulates the registration, accreditation, and endorsement of a range of health professions in Australia, including psychology (Australian Health Practitioner Regulation Agency, 2017a,b,c).

AHPRA’s Register publicly lists the names of health practitioners who practice within the scope of their registration. A separate Register for Canceled Practitioners includes those health practitioners who have been served an undertaking not to practice. Psychologists listed on the Register for Canceled Practitioners were excluded from the study because the researchers wanted to recruit clinical psychologists who were at the time practicing. Similarly, for ethical reasons, psychologists were not invited to participate in the study if the Psychology Board of Australia (2011) had reprimanded them due to the chastisement of conduct or formal rebuke. Relevant psychologists were forwarded by email a cover letter, participant information sheet, and consent form.

The psychologists selected had indicated verbally that they had experienced burnout according to Maslach and Jackson’s (1981a) MBI 22 attitude and feeling statements. Maslach and Jackson’s construct of burnout is both internally valid and reliable. Those researchers report reliabilities of 0.90 for emotional exhaustion, 0.79 for depersonalization, and 0.71 for personal accomplishment. The MBI is externally valid and is based on the data collected from a wide range of different health professionals, including, psychologists, psychiatrists, psychotherapists, and counselors. Maslach and Jackson (1981b) confirm that the measure has a high degree of convergent validity. Those researchers had independent assessors corroborate the self-ratings of health professionals, who also observed the behaviors of those professionals. Some of the questions that they asked health professionals included: “How drained did you feel?” “How did you respond to your clients?” “Were you: upset, anxious, or exhausted?”

MBI, statements were modified from the first person point of view to the second person point of view. Those statements were made appropriate to be asked to the participants, for example, the statement “I feel like I am at the end of my rope” was changed to: “When you experienced burnout, did you feel like you were at the end of your rope?” To have experienced burnout, participants needed to agree to at least half of the statements for emotional exhaustion and depersonalization. As the MBI was negatively scaled for the dimension of personal accomplishment, participants needed to disagree with at least half of those statements. Since explicit declarative memories deteriorate over time (Ward et al., 2013), clinical psychologists were only invited to participate in the study if they had experienced burnout recently, that is, no more than 2 years before the study commenced. This was particularly important for the validity of the present study, as participants were asked to consciously remember and recall aspects of their experiences when they were burnt-out. Participants answered the MBI modified questions prior to engaging in telephone interviews.

Six separate in-depth semi-structured interviews were conducted to look for commonalities in clinical psychologists’ experiences of burnout. The small number of participants was appropriate for the present study as data saturation appeared to have been achieved. Indeed, Guest et al. (2006) noted that data saturation may be attained by as little as six interviews. Data saturation is reached when there is enough information to replicate the study (O’Reilly and Parker, 2012), when the inability to obtain additional new information has been attained, and when further coding is no longer feasible (Guest et al., 2006). At the time of being interviewed over the telephone, all psychologists were employed in the private sector and they also ran solo practices. The demographic information collected for each participant is outlined in **Table 1**.

**Table 1:** The key demographic data of the Australian clinical psychologists who engaged in semi-structured interviews.

Participant	Sex^1^	Age	Highest	State	Marital status^2^	Ethnicity^3^	Direct client	Months of	Years	Years endorsed
		(years)	educational				contact hours	burnout	registered as a	to practice
			attainment				(per week)^4^		psychologist	clinical psychology
1	F	48	Masters	Western Australia	Never married	NWE	28	7	16	14
2	F	29	Doctorate	Queensland	Never married	NWE	26	6	7	4
3	F	41	Masters	Victoria	Married	SEE	20	4	10	4
4	F	54	Masters	New South Wales	Married	NWE	18	12	20	8
5	M	36	Doctorate	New South Wales	Married	SEE	26	9	10	6
6	M	72	Doctorate	New South Wales	Married	SEE	23	3	25	25

*^1^Sex refers to a person’s biological category as being either male (M) or female (F) (Australian Bureau of Statistics [ABS], 2011a). ^2^Marital status formally classifies a person’s living arrangements as never married, widowed, divorced, separated, married, or not applicable (Australian Bureau of Statistics [ABS], 2011b). ^3^Ethnicity refers to the broad geographical areas where culture and ethnic groups originally developed (Australian Bureau of Statistics [ABS], 2011c). Ethnic categories include: Oceanian (O), North-West European (NWE), Southern and Eastern European (SEE), North African and Middle Eastern (NAfME), South-East Asian (SEA), North-East Asian (NEA), Southern and Central Asian (SCA), People of the Americas (Am) and Sub-Saharan African (SSAf). ^4^Direct client contact hours refer to the estimated or average hours that therapists’ directly spend performing specific tasks (i.e., psychological assessment, intervention, prevention, consultation and management planning) with their clients in any given week (Psychology Board of Australia, 2011).*

### Procedure and Materials

Semi-structured interviews were conducted with the following research question in mind: “What are clinical psychologists’ experiences of burnout?” All interviews were digitally audio-recorded in the year 2015. The interviews lasted for 35–49 min and an average of 42 min. The question was framed based on the premise that there was very limited published research regarding clinically practicing psychologists’ experiences of burnout. A brief interview guide was developed, which outlined the initial structure of each interview, including information about the researchers, their university affiliation, the purpose of conducting the interview, and the construct of burnout conceptualized by Maslach and Jackson (1981a). All clinical psychologists who were interviewed met the definition and criteria of burnout published in Maslach and Jackson’s original “Research Edition Manual.” Given the sensitive nature of the topic of burnout, information regarding ‘Lifeline,’ was provided to participants. Lifeline (2017) is a fee-free Australian counseling and support service that provides access to “24 hour crisis support and suicide prevention services” for adults.

Participants were reminded that they could withdraw from the study at any time without reason by requesting for the interview to cease. After they were asked the open-ended question, “what are your experiences of burnout?”, conversations individually evolved. The researchers were an ‘instrument’ who listened carefully and were responsive to the participants’ elaborated experiences. In line with earlier researchers (Gillham, 2000; Seidman, 2006; Minichiello et al., 2008; Hennink et al., 2011), the present researchers demonstrated respect and understanding by asking follow-up questions, by using a variety of rapport-building strategies to make participants feel at ease, and by building trust. The clinical psychologists were provided with opportunities to describe in rich detail their experiences of burnout. As Patton (2002) puts it, researchers can comprehend participants’ experiences by experiencing them through their language by asking questions. For example, clinical psychologists were empowered to direct the conversation and to elaborate on their own subjective experiences of burnout.

Participants were encouraged to expand on their responses through the researchers use of several questioning techniques, as per Kvale and Flick (2007) and Brinkmann and Kvale (2015), including: (1) Introductory questions to introduce the topic of burnout, for example, “What are your experiences of burnout?” (2) Specifying questions to obtain more information, for example, “…think of any particular experiences you have had, which were kind of the turning point of you wanting to change something about yourself or the way you were doing things at work to overcome the burnout?” (3) Probing questions to follow up what has been said and to get more detail, for example, “You mentioned earlier that you found it quite difficult to overcome challenging aspects in your role as a clinical psychologist and umm you had difficulties focusing… Maybe you could expand on that and talk about how you managed your workload and how you managed dealing with clients?” (4) Direct questions to obtain specific details, for example, “how long was the period of treatment with the client?” and “Was that happening at the same time your wife was in hospital?” (5) Structuring questions to move the interview onto the next subject, for example, “Yeah, yeah. I’m interested in hearing more about the coping mechanisms there and ummm how you could focus specifically on the job?” (6) Interpreting questions to ensure that the researchers were on track, for example, “from what you’re telling me, your burnout resulted in elevated levels of stress and anxiety, is that correct?” (7) Silence and pauses to encourage participants to answer questions, for example, “Ok. That makes sense [pause]. The participant responded: … Well, you’ve got-to because otherwise you don’t – you can’t keep working in your field.”

The researchers’ prior engagement with the clinical psychologists before the interviews provided additional context regarding their lived experiences. Throughout the interview and data analysis process, the researchers engaged in reflexivity, which Finlay and Gough (2003) describe as a process of self-awareness, whereby researchers think about how they form understandings and make decisions regarding the study.

### Qualitative Data Analysis

Data were de-identified before conducting formal exploratory analyses to protect the privacy and confidentiality of the clinical psychologists (Stebbins, 2001). Pseudonyms were used when transcribing the data and no identifiable information was used in Hammond’s (2015) thesis. Data analyses commenced when interview recordings were played back, listened to for meaning, and transcribed verbatim, as per Brinkmann and Kvale’s (2015) guidelines. There was complete data immersion and an accurate understanding of the context and the experience of each clinical psychologist. Data were approached with flexibility, an open mind, and a reflexive stance. Orthographic transcription provided a rigorous and thorough verbatim account of verbal and non-verbal utterances (for example, coughs, pauses, umms, etc.) (Braun and Clarke, 2006).

Interviewing clinical psychologists one-on-one provided a transparent means of understanding their individual lived experiences. The method ensured that the ‘voices’ of the clinical psychologists interviewed were kept without abstracting their viewpoint out through analysis. Conducting confidential semi-structured interviews allowed the researchers to capture the reactions and behaviors that were included in the data, in addition to the thoughts, impressions, feelings, interpretations, and understandings of the clinical psychologists’ experiences. The use of open-ended questions provided clinical psychologists multiple opportunities to verbally describe their experiences, as they related to what it was like to experience occupational burnout.

**FIGURE 1:**
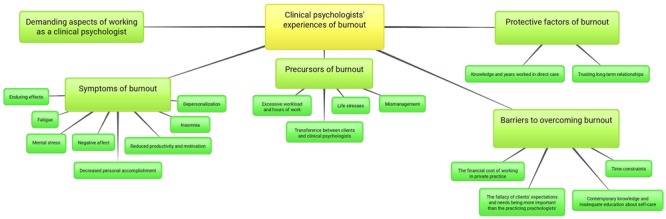
A thematic map illustrating the 5 key themes and sub-themes illuminated from thematic data analyses of clinical psychologists’ experiences of burnout.

Braun and Clarke’s (2006, p. 96) method and “15-point checklist” for “good thematic analysis” were used to analyze the data, to code the data, and to identify emerging themes. The researchers:

1 *Became familiar with the experiences of each clinical psychologist by immersing themselves in the data*. Interviews were carefully listened to and initial notes regarding the meaning of the text were made before being transcribed. The passages of orthographic transcripts were actively read several times and were searched for meanings and patterns.
2 *Systematically generated initial codes and collated relevant data to each code by using NVivo* (Version 10.1.01 for Mac). Initial themes were identified and coded semantically based on their explicit meaning and annotations and memos were made to help understand the meaning of the data. The open coding process resulted in 404 lower-level first-order constructs for clinical psychologists’ transcripts. Sequences of text were attached to the transcribed data to describe, name, and classify (Strauss and Corbin, 1990) clinical psychologists’ experiences of burnout. Data immersion was suspended for 2 weeks on two separate occasions to allow for data crystallization.
3 *Searched for themes by gathering data and collating codes into themes.* The process of axial coding was followed to analyze individual datum (Flick, 2009) and the following questions were asked: (1) What were the issues the clinical psychologist experienced with his/her burnout? (2) Who were the actors involved in their experience of burnout and what roles did they play? (3) How did burnout first emerge? (4) When did the clinical psychologist’s experience of burnout occur? (5) For what length of time did the clinical psychologist experience burnout? (6) Where did the clinical psychologist experience burnout? (7) What was the level of intensity of the burnout? (8) Why did the clinical psychologist think that burnout occurred? Commensurate with Strauss and Corbin (1990), axial coding involved refining nodes according to specific boundaries and relating codes to each other. In line with Ryan and Bernard (2003), themes were identified through repetitions, indigenous categories, metaphors, analogies, transitions, similarities and missing data.
4 *Reviewed themes* by checking that the themes worked in relation to the coded extracts and the entire data set and by generating a branching hierarchical tree in NVivo and then developing the final thematic map of the analysis shown in **Figure 1**.
5 *Defined and named themes* by redefining the specific features of each theme including labels and definitions that represent the experience of each clinical psychologist using a codebook (Supplementary Tables 1–5) (DeCuir-Gunby et al., 2011).
6 *Developed a thesis and then produced the present scholarly article* by selecting vivid and compelling quotations and by relating the analysis to the research question and literature.

## Findings

In order of prominence, five main themes were identified both across the preponderance of data and within transcripts, including demanding aspects of working as a clinical psychologist and symptoms, precursors, protective factors, and barriers to overcoming burnout. These themes and subthemes are shown graphically in **Figure 1**.

### Demanding Aspects of Working as a Clinical Psychologist

All psychologists experienced taxing and intensive jobs that required additional effort and skills to manage. One participant outlined the negative impact of inadequate education on job performance: “They didn’t tell us anything about burnout… so I didn’t even think it was something that happened, but I know it happens to a lot of my colleagues…” The psychologist’s classmates did not learn about self-care when they studied at university, so they may not have been equipped to meet clients’ needs. Some of the psychologists interviewed suggested that their lack of skills-based training appears to be a major problem when practicing clinical psychology.

### Symptoms of Burnout

Three of the clinical psychologists interviewed described having several symptoms that appear to be related to burnout. This finding is not surprising given that decreased personal accomplishment is like reduced productivity and reduced motivation and emotional exhaustion is related to fatigue. Given that the study was inductive rather than confirmatory, the small number of participants with the full list of burnout symptoms is not considered to be a limitation. As outlined earlier, the clinical psychologists who were interviewed met more than half of the criteria for burnout according to the MBI (Maslach and Jackson, 1981a). Despite that emotional exhaustion was not identified as a symptom, psychologists identified a broad range of symptoms of burnout, including enduring effects, mental stress, fatigue, decreased personal accomplishment, negative affect, depersonalization, reduced productivity and motivation, and insomnia.

#### Enduring Effects

Five psychologists had persistent burnout. Participant 3 described having existential problems, including, fluctuating moods, high workload, and mental stress:

It’s happened a couple of times over the last 5 years… the second time it’s really come up was um, was just recently… I think I’m in recovery at the moment… I’m definitely still feeling burnt-out about those insurance cases… I’m trying to cut back on that work…

#### Mental Stress

Four psychologists experienced mental stress. Participant 1 felt stressed leading up to a holiday and felt guilty taking a break from work. Internal factors encouraged the psychologist to take a break and acted as a conduit to alleviate mental stress:

… There was lots of qualms and pressures. It really was [stressful], and that said to me that I might have been carrying that stress for such [deeper tone and emphasis] a long time and it had probably been 18 months/ 2 years… before we actually had a break.

#### Fatigue

Four psychologists had intense feelings of fatigue. Participant 2 indicated that there was a relationship between fatigue and emotional exhaustion: “… I felt very tired… just exhausted I remember… by the time I got to my Christmas break I was pretty fried–pretty frazzled.” The psychologist learned from previous experiences, which suggests that one’s past may prevent one from pushing one’s self to the limit due to fatigue.

#### Decreased Personal Accomplishment

Participant 5 described reduced engagement and seeking distractions from work: “… If I had a break, I would just drop everything… maybe surf the internet, look at the news, or do something completely unrelated to my work.” The psychologist used habitual strategies to escape from daily stressors.

#### Negative Affect

Three psychologists described the impact of negative affect on their work. Participant 4 identified a spiral effect, whereby negative thoughts had an impact on job performance and mental health: “… I think the whole burnout thing impacted on my mood ‘cos I knew what I had before me… that then leads to negative thinking about yourself…”

#### Depersonalization

Participant 5 described depersonalization and that it is common for psychologists to think cynically about their clients: “What contributes to burnout is seeing clients who push your buttons… There are always some clients where I feel some dread about seeing them…” The psychologist blamed clients for having those feelings and used the term “normalizing” to highlight that client disengagement is common. The psychologist believed that unethical behavior is part of the culture of clinical practice because therapists avoid admitting when they need professional help.

… Regarding the focus, in particular... I mentioned that to colleagues, but they all kind of knew what I was talking about. That I was normalizing… That happens to all of us.

#### Reduced Productivity and Motivation

Three psychologists had reduced productivity and motivation to engage in activities because it was harder to achieve positive outcomes at work. Participant 2 was not interested in work and made excuses to avoid going to work. The psychologist’s state-of-mind had consequences for the well-being of clients and conflicted with the Australian Psychological Society’s (2007) Code of Ethics:

… One of the main things I remember is driving to work… just wishing I felt sick that day or, you know, that I get a flat tyre or something just so I didn’t have to go.

#### Insomnia

Two clinicians had sleeping problems and had an understanding regarding the symptoms of burnout, including an understanding about sleep deprivation and moods. Earlier discussions with participant 4 demonstrated her desire to avoid future similar experiences:

… I hadn’t been sleeping well… I think it was probably 12/18 months. We know all the difficulties that occur when we’re not sleeping right, you know, mood and concentration…

### Precursors of Burnout

Physical and mental precursors of burnout include excessive workload and hours of work, life stresses, mismanagement, and transference between clients and clinical psychologists.

#### Excessive Workload and Hours of Work

All participants described excessive workload and hours of work as being the main precursors to burnout. Participant 5 described working 44 h per week:

… I was working… a Monday afternoon from twelve (12 noon) to eight (8.00 pm). Tuesday morning from seven (7.00 am) … my boundaries were pushing that out ‘til five (5.00 pm). On Wednesday I would have supervision in the morning and then start seeing clients at 12 (12 noon) and finish at 8 (8.00 pm) … Thursday I would see clients at 12 (12 midday) and finish at 8 (8.00 pm). Friday I would see clients from 7 (7.00 am) … and finish… at 5 (5.00 pm) but those were ridiculous hours… there were a few days where I’d do late nights and come in early morning… and that was just too much.

The psychologist made an error in judgment and outlined the lack of control that therapists have over their work hours when managing their practices. Like other psychologists, the participant had habitually worked additional hours, which had become institutionalized over time.

#### Life Stresses

Five psychologists suggested that everyday stressors result in heightened stress. Participant 2 described the impact of two stressors on burnout, namely, lack of income stability and academic pressures: “…You’re never really working full-time as you’re doing uni–well, that’s what I was doing anyway... It was a professional doctorate, so I wrote about three papers.” Another stressor highlighted was the psychologist’s lack of support from a previous clinical supervisor. The psychologist’s previous supervisor did not conform to the norm of health practitioners, who are supportive and encourage ongoing staff development:

… I didn’t really have the greatest professional support… If you’ve got a good supervisor that you can trust, that’s always nice. My other supervisor in that burnout year was just very ‘Suck it up. This is what it’s like to be a psychologist’...

Life stresses had an adverse impact on the psychologist’s social interactions, “I really think it just [pause] made all those other factors harder to deal with, so, if there was any relationship stress… anyone sick. That was a lot harder.”

#### Mismanagement

Four psychologists outlined problems with managing workload. Participant 6 highlighted that mismanagement led to personal incompetence. Based on the rich contextual information provided, the clinician spoke in third-person to feel more comfortable with revisiting events. The psychologist highlighted how firsthand experiences result in negative social impacts:

A complaint arose because [a clinical psychologist] did an assessment on a nineteen-year-old boy who had Asperger’s and the parents reported him because he got the date of birth wrong… he misquoted the parents, and the reason is he was driving. The trolley car already went over the cusp and was hurtling into the abyss.

#### Transference between Clients and Clinical Psychologists

Four psychologists indicated that they experience transference of emotions and attitudes between them and their patients. Psychologists are constantly interacting with clients and are prone to their negative dispositions. Their mixed caseloads often consist of people who have severe mental health problems. Participant 3 was clearly emotionally attached to clients.

… There’re a few people that are fairly high functioning and come in with clinical levels of anxiety and depression… There’s a couple of um–quite clinically severe cases… I’ve got one or two patients with eating disorders… It’s very intense.

### Protective Factors of Burnout

Protective factors reduce the likelihood of people experiencing mental health problems. In this study, clinical psychologists highlighted protective factors of burnout, which included knowledge and years worked in direct care and trusting long-term relationships.

#### Knowledge and Years Worked in Direct Care

Five psychologists suggested that knowledge of their roles and experience with clients protected them from burnout. Participant 4 indicated that engaged psychologists are less prone to having burnout: “… the amount of enjoyment and meaning that psychologists were getting out of work. One client actually said to me: ‘Look, I don’t even know what burnout is’…” Psychologists’ agency means that they make individual decisions regarding their clients. Positive interactions between clinical psychologists and their clients can be engaging and thought-provoking.

#### Trusting Long-Term Relationships

Five psychologists described trusting and long-term relationships. Participant 1 outlined the importance of supervisor-therapist relationships:

The supervisor I had–um for my registration was actually just one that was in the workplace. Um, I was very fortunate to have worked in a hospital-based system where there was a senior clinical psychologist, and he took on my supervision.

The psychologist described having negative experiences with a previous supervisor and made comparisons to the then current supervisor: “... If there’s something going on for you that’s affecting your work, they’ll [supervisors] be able to handle it without, sort of, shaming you, I think.”

### Barriers to Overcoming Burnout

Barriers to overcoming burnout include the fallacy of clients’ expectations and needs being more important than the practicing psychologists’, the financial cost of working in private practice, contemporary knowledge and inadequate education of self-care, and time constraints. Those factors interfered with clinical psychologists’ abilities to overcome burnout.

#### The Fallacy of Clients’ Expectations and Needs Being More Important than the Practicing Psychologists’

All participants suggested that their clients’ expectations and needs were more important than their own. Participant 6 described the difficulty of meeting peoples’ expectations:

… You do well, and people stand up and applaud you, but do one thing wrong and your reputation is shattered, and once that happens… It’s really very difficult to get back on track. We’re expected to be strong, and we’re expected to be informed and professional.

The clinical psychologists who were interviewed indicated that the perspectives of their peers and their clients exert pressure on them to perform well, avoid mistakes at all costs, learn from their experiences, and maintain professional relationships. Participant 6 justified the importance of ‘duty of care’ to clients: “… You can have people you’re working with whom you have concerns. I’ve had to say: ‘Look, I’m gonna refer you to a psychiatrist for admission’…” The psychologist made use of traditional referral processes, which involve referring severe clients onto specialized health practitioners and psychiatrists.

#### The Financial Cost of Working in Private Practice

Five psychologists indicated that being predominantly employed in private practice results in high financial costs. ‘Costs’ not only refer to a sum of money that is paid or spent to buy something, but also opportunity costs, which are those costs associated with foregoing something. Participant 2 indicated that the cost of working in private practice led to not going on a holiday: “…It was the whole um, so, ‘I’m at uni I’m so poor, keep working’ ha ha… kind of situation.” The psychologist could not escape work stressors to take a holiday, which is concerning because therapists are continuing to work, despite feeling burnt-out and exhausted.

#### Contemporary Knowledge and Inadequate Education About Self-Care

Four psychologists mentioned that their contemporary knowledge regarding self-care and insufficient education were barriers to overcoming burnout. Participant 3 highlighted not being aware of the most common definition of burnout (Maslach and Jackson, 1981a): “I dunno ha ha [laughs]. I haven’t read about burnout specifically for years!” Like the other psychologists who were interviewed, participant 3 found it difficult to identify relevant published findings, which could have impacted her ability to provide professional client care.

#### Time Constraints

Three psychologists felt limited given the time they had to complete everyday tasks. Participant 3 said that there was “… probably just not enough um, like personal time out to reflect and take stock of things.” Putting adequate time aside for mental health reasons is important, not just for psychologists, but also for other health practitioners. The psychologist found it difficult to maintain an exercise routine going to a local gym, and could not release built-up tension to maintain good health: “… I found going to the gym quite time-consuming.”

## Discussion

The key research question was “what are clinical psychologists’ experiences of burnout?” Most of the psychologists who were interviewed in the study suggested that working as a psychologist is highly demanding and that burnout has a variety of symptoms, including enduring effects, mental stress, fatigue, decreased personal accomplishment, negative affect, depersonalization, reduced productivity and motivation, and insomnia. Some psychologists in the study highlighted that burnout precursors include excessive workload and hours of work, life stresses, mismanagement, and transference between clients’ and their psychologists. Protective factors of burnout include the psychologists’ knowledge and years worked in direct care and trusting and long-term relationships. Further, psychologists in the study suggested that barriers to overcoming burnout are the fallacy of clients’ expectations and needs being more important than the practicing psychologists’, the financial cost of working in private practice, contemporary knowledge and inadequate education about self-care, and time constraints.

Each of the major themes will now be explored in turn and set in the context of published research findings. The theme ‘demanding aspects of working as a clinical psychologist’ is commensurate with Maslach’s (1982) burnout model, Huberty and Huebner’s (1988) findings and more recent quantitative research published by Lee et al. (2011). The burnout model suggests that interpersonal demands at work, that is, stressful working conditions, lead to burnout. Huberty and Huebner indicate that work demands lead to increased emotional exhaustion. Similarly, Lee et al.’s (2011) meta-analysis of psychotherapists identifies a moderate relationship between over involvement and emotional exhaustion. Huebner (1993, 1994) identifies relationships between job functions and burnout. Job functions are like interpersonal demands at work, which was a key theme identified in the present study. Conversely, Farber and Heifetz’s (1982) infer that non-reciprocated attentiveness and constant giving of oneself is demanding. Those themes were not identified in the present study.

The most prominent sub-theme of ‘symptoms of burnout’ was that burnout has ‘enduring’ effects. That sub-theme was not identified in other published research, possibly due to the paucity of qualitative research about clinical psychologists and burnout. In the present study, clinicians indicated that their burnout persisted and that they had recurring episodes. Treatment options and strategies to overcome burnout may, therefore, be ineffective or at best, short lasting.

The theme ‘precursors of burnout’ consisted of two principal sub-themes, namely, excessive workload and hours of work, and life stresses. The former sub-theme was not surprising given that earlier researchers (Farber and Heifetz, 1982; Maslach and Leiter, 1997) identify direct relationships between clinical psychologists’ burnout, overworking, and dealing with clients’ problems. Lee et al. (2011) also report that over involvement, which is like excessive workload stems from emotional exhaustion. Furthermore, Oser et al. (2013) identify that large caseloads and excessive paperwork lead to burnout of substance abuse treatment counselors. Those sub-themes are commensurate with the interpersonal demands aspect of the burnout model (Maslach, 1982). Life stresses could include many stressors, which have previously been identified by school psychologists (Huebner, 1992), for example, conflict, lack of resources, and public speaking. In an earlier study, Huberty and Huebner (1988) identify that external pressures, for instance, facilities and travel, result in stress and burnout. Those findings are not dissimilar to the findings outlined in this study.

The sub-themes of ‘protective factors of burnout’ were knowledge and years worked in direct care and trusting long-term relationships. The present study is the first to be published in a peer-reviewed journal that identifies that knowledge and years worked in direct care protect clinical psychologists from burnout. Huebner (1992) and Maslach and Leiter (1997) identify relationships as being important in protecting clinical psychologists from burnout. Some of the therapists interviewed in the present study indicated that they were more prone to burnout if their supervisors or people close to them fail to provide adequate support. Similarly, McCormack et al. (2015) found that social support has a positive influence on burnout, which aligns with the theme of trusting long-term relationships in the present study. Oser et al. (2013) highlight that co-worker support and clinical supervision are influential factors to consider when preventing burnout in counselors. Clearly, trust and rapport are key factors that maintain effective relationships; they encourage health practitioners to discuss their problems and to develop appropriate solutions to overcome burnout.

Two sub-themes emerged regarding ‘barriers to overcoming burnout,’ namely, the fallacy of clients’ expectations and needs being more important than the practicing psychologists’, and the financial cost of working in private practice. Published research has not yet identified those barriers to overcoming therapist burnout, however, we offer some possible explanations. The clinical psychologists who were interviewed in our study suggested that society has an expectation for psychologists to assist people with their problems. If this is indeed correct, psychologists may feel sub-servant to the needs of their clients, which could directly have an impact on the health of therapists. Further, the principle of confidentiality makes it incredibly difficult to trace the experiences of clinical psychologists and their clients over time. One would therefore find it difficult to analyze the assumptions psychologists make about themselves and the expectations that they have for each other about therapeutic relationships.

There is scope to conduct a cross-sectional online study to identify the perceptions and assumptions that clinical psychologists and clients have about burnout and each other. Broader literature regarding health practitioners who work in private practice (for example, medical practitioners, chiropractors, dentists, and optometrists) could highlight commonalities between professions. For instance, do most of those health practitioners experience financial problems in their private practices, and if so, does that have an impact on their burnout? What perceptions do these broader range of health practitioners have about themselves and their clients, and vice versa? Published literature regarding the above four types of health practitioners may shed light on these questions and is worthy of further investigation.

### Strengths with the Study

Interviews with clinical psychologists provided contextual information about their experiences of burnout. Those discussions led to rich descriptions of psychologists’ experiences, which would have some transferability across employment settings, employment sectors, industries, and roles. The semi-structured interviews enhanced researcher-participant rapport and allowed for systematic analyses of transcribed data. In line with Creswell’s (2014) suggestions, data saturation appeared to be reached by the fifth interview as no further codes emerged regarding participants’ experiences. Additional research consisting of greater numbers of clinical psychologists may confirm that data saturation can be reached with a small number of participants. Although psychologists were difficult to recruit, they were comfortable with openly and honestly discussing their experiences. Psychologists were difficult to recruit because those who have experienced burnout found it difficult admitting that fact and were concerned about their professional reputation.

Memos were regularly made and a research diary was updated at each stage of data analysis. That allowed the researchers to identify their subjective perspectives, biases, vested interests, cultural factors, and assumptions that could influence how they viewed the study’s data (Finlay and Gough, 2003). There was a deep understanding about burnout for the six clinical psychologists who were interviewed, which occurred at different times, between various employment settings, and within different contexts. Peer debriefing among the three authors about the study provided opportunities for feedback, particularly when a codebook was developed (DeCuir-Gunby et al., 2011) and ideas were expressed. Key points of discussion included methodological issues and the strategies used to prevent ethical problems and reduce harm to the participants. The psychologists reviewed their transcripts and identified some minor misconceptions, which were later clarified and confirmed. An audit trail shows that the study is dependable, trustworthy, and that the present authors were reflexive in their approach.

### Limitations of the Study

The study is limited to descriptive analyses of the commonalities between Australian clinical psychologists’ experiences of burnout. The present study does not, nor should it be expected to, provide statistical data that could be used to generalize to the wider population of psychologists. Indeed, another qualitative or a mixed-methods study with a larger sample size may provide further information about clinical psychologists’ experiences of burnout. Purposive sampling lacks the randomness of probability sampling of quantitative research, which is required to generalize the data (Hennink et al., 2011).

As with all qualitative research (Starks and Trinidad, 2007), the present authors knowingly and unconsciously influenced the data through their personal biases and opinions. Leading up to and during semi-structured interviews, clinical psychologists could have reacted differently, for example, they may have filtered their thoughts or misrepresented their experiences. Through engaging in proven interview techniques, reflexivity, developing rapport, and making participants feel at ease, the researchers mitigated the effects of demand characteristics and encouraged natural and free-flowing discussions. However, the researchers had both a conscious and unconscious influence on how they interpreted audio recordings, transcribed data, and analyzed data.

### Implications

The present study’s findings may benefit the personal practices of a variety of health professionals across multiple industries. By being well-informed about burnout’s risk factors and precursors, health practitioners may adapt their work styles to prevent the occurrence of burnout and enhance their adaptive coping strategies. Clinical psychologists and related health professionals may be more aware of burnout’s early warning signs and symptoms. Therapists may be better placed to take appropriate actions to prevent the severity of burnout increasing. If health practitioners get burnt-out, the present study could assist to identify barriers to treatment that would allow them to seek out help.

Evidence from the present study may benefit professional practice and international communities. Health professionals may transfer this study’s findings, to some degree, across professions, across different industries and within the profession of clinical psychology. Potential benefits to professional practice include reduced sick leave, greater staff engagement, enhanced productivity at work, decreased resignation of health and community services industry staff, and lower mental health rehabilitation costs.

There are opportunities for universities and colleges to restructure clinical psychology educational programs to include additional modules about self-care. Individuals who seek assistance from health professionals would be more likely to ask for help earlier. Application of the present research findings would improve client-therapist relationships, which would enhance the communities’ expectations and understanding of mental health. Additional flow-on effects for families, employers, colleagues and the community could result in richer and more positive work and home environments.

Future qualitative research could build on this study’s findings and enhance transferability between contexts by recruiting a larger number of clinical psychologists employed in several industries. Researchers could perform a large-scale content analysis of open-ended interview data of psychologists’ burnout experiences. Content analyses would allow researchers to easily identify recurring patterns and themes within the data. That information would inform health practitioners regarding burnout and the availability of efficacious treatments.

## Conclusion

The findings presented in the present study demonstrate the value of conducting descriptive qualitative research. Clearly, the experiences of clinical psychologists need to be contextualized rather than individualized to understand burnout. The key findings of the present study were that burnout has a variety of symptoms, for example, its enduring effects, mental stress, fatigue, decreased personal accomplishment, negative affect, depersonalization, reduced productivity and motivation, and insomnia. Precursors of burnout include excessive workload and hours of work, life stresses, mismanaged workload, and transference. Knowledge and years worked in direct care and trusting long-term relationships are protective factors. Barriers to overcoming burnout include misconstrued expectations about one’s clients and finances, contemporary knowledge and inadequate education regarding self-care, and time constraints.

There remains a gap in recognizing and understanding the lives of clinicians who have had burnout. Upon reflecting about the present study, we considered conducting a larger mixed-methods study consisting of different types of health professionals to identify key differences regarding their burnout experiences. The positive implications of burnout research would be personally and professionally rewarding for health practitioners across numerous industries. Findings would reduce burnout, lower absenteeism rates, and lessen the strain placed upon the provision of healthcare.

## Access To The Data

TH may provide de-identified data for the present study by email: thammond@csu.edu.au. Interested stakeholders may communicate by email in the first instance with TH and then AC and SD.

## Author Contributions

TH primarily designed the study, developed the main research question, recruited the clinical psychologists who participated in the study, and collected the data. All the researchers analyzed the data, reviewed and revised the work for intellectual content, and drafted the manuscript for publication. SD was the primary supervisor of the honors project and AC was the co-supervisor of the honors project. SD and AC contributed to the conception and design of the study and reviewed and revised the manuscript. They provided feedback regarding the choice of analysis, ethical considerations, and critically reviewed the manuscript prior to being submitted to the journal “Frontiers in Psychology.” All the authors have approved the final version of the manuscript to be published and have agreed to be accountable for all aspects of the work.

## Funding and In-Kind Support

The combined sum of $7,000 (Australian dollars) from Charles Sturt University, in the form of a “Foundation Honors Scholarship” (grant 18548), “Honors Scholarship” (grant 10272), and “Student Contribution Fee Exempt Award” (grant 14132) was managed and reconciled by TH. TH notified the co-authors in writing regarding the outcomes of each scholarship application. The authors give thanks to Frontiers in Psychology, which provided in-kind support in the form of a full fee-waiver to the value of $2,490 (United States dollars), grant 2016-0073254-9.

## Conflict of Interest Statement

The authors declare that the research was conducted in the absence of any commercial or financial relationships that could be construed as a potential conflict of interest.
