# Supplementary material for: A Thematic Inquiry into the Burnout Experience of Australian Solo-Practicing Clinical Psychologists
Source: Front Psychol. 2018 Jan 19;8:1996. doi: 10.3389/fpsyg.2017.01996 (PMC5785720; doi:10.3389/fpsyg.2017.01996)
Supplement: Supplementary file 1 [file Table_1.docx]

Supplementary Material

A Thematic Inquiry into the Burnout Experience of Australian Solo-Practicing Clinical Psychologists

Trent. E. Hammond*, Andrew Crowther, Sally Drummond

*** Correspondence:** Mr Trent Hammond: thammond@csu.edu.au

# Supplementary Tables 1–5

The present supplementary material contains a codebook, which clearly identifies the first author’s reasons for assigning individual codes to particular themes in the study (Hammond, 2015). DeCuir-Gunby et al. (2011, p.138) define a codebook as “a set of codes, definitions, and examples used as a guide to help analyze interview data.” The codebook allowed for systematic coding and increased the credibility of coding.

The codebook includes labels for easy identification, precise and accurate definitions, descriptions, relevant inclusion and exclusion criteria, and example quotations. Themes are exhaustive yet mutually exclusive– all data fits into only one category, sensitive to datum content, and conceptually congruent– the same level of abstraction characterizes all classes at the same degree. The codebook identifies relevant codes that apply to the research question: “What are clinical psychologists’ experiences of burnout?”

| Key Definitions  Demanding: To require great patience, skill, calling for intensive effort or attention or taxing efforts. | | |
| --- | --- | --- |
| Sub-Theme Label | **Definition/Description** | **Example Quotation/s From the Raw Data** |
| The first author did not identify any sub-themes for the theme ‘demanding aspects of working as a clinical psychologist.’ | Datum should be coded to this theme if the psychologist:   - describes demanding aspects of his/her role, - outlines demanding aspects in general or - specifically identifies challenging aspects of someone else, who is also a clinical psychologist.   Datum should not be coded to this theme if the psychologist describes stressors related to his/her position in general. In that case, the topic of life stresses may be more appropriate. | Umm, and I was sort of wishing for what I was calling kind of a normal job... But, umm because this job doesn’t feel normal at all. Even at the best of times, it’s not normal. In a good way, sometimes and in a not so good way at other times. But, it’s not usual to sit with people in distress for so many hours in a week (Participant 5).  I think, in the area where my practice is based–it’s um–it’s an area that’s known for having quite a lot of psychosocial vulnerability–low socioeconomic status (Participant 3). |

## Table 1. Demanding Aspects of Working as a Clinical Psychologist.

## Table 2. Symptoms of Burnout.

| Key Definitions  Symptom: A change in the physical or mental condition of a person and a sign of the existence of something. | | |
| --- | --- | --- |
| Sub-Theme Label | **Definition/Description** | **Example Quotation/s From the Raw Data** |
| Enduring Effects | Burnout continues for an extended period or it is long-lasting. This theme specifically relates to burnout, as opposed to other symptoms or conditions. | ... It did take quite a while to be fully back on my game. I think that six-month-period is really… a critical number (Participant 1). |
| Mental Stress | Mental stress is an adverse reaction experienced by clinical psychologists when workplace demands and responsibilities are greater than what they can comfortably manage, or are beyond their capabilities. Datum should not be coded as ‘mental stress’ if psychologists suggest that any of the following factors directly resulted in stress:   - decreased personal accomplishment, - adverse effects, - fatigue, - negative affect, - depersonalization, - insomnia, - reduced productivity, - motivation and - the enduring effects of burnout. | So rather than move towards it [complete relevant paperwork] and get on top of things I was moving away from it, which meant that it was also getting on top of me... It wasn’t unconscious. It was just something I found myself doing... I would just sit on my chair and sometimes I’d shut my door. I didn’t want to see anyone. (Participant 5). |
| Fatigue | Fatigue refers to weariness or exhaustion that generally results from physical work, mental work or sleep deprivation. | There was a whole lot of other stuff going on in my life and what I started to notice was, um, well exhaustion... I guess I was just getting exhausted (Participant 4). |
| Decreased Personal Accomplishment | Reduced personal accomplishment is the perception that one’s accomplishments on-the-job fall short of personal expectations. It may involve negative self-evaluation (Maslach and Jackson, 1982). | ... I think it was the second year of my doctorate I was thinking about pulling out... So, it was really kind of, and the first assignment that I handed in for my doctorate was–I got a really bad mark, and it was like: ‘What? I’ve never experienced that before. (Participant 4). |
| Negative Affect | Negative affect refers to negative emotions (for example, nervousness, tension and worry) and poor self-concept. The first author excluded Major Depressive Disorder and other mental disorders described in the Diagnostic and Statistical Manual of Mental Disorders (DSM-V) (American Psychiatric Association, 2013). This sub-theme may contain datum of clinical psychologists, who describe their symptoms that relate to one or more mental disorders in the DSM-V. For example, a depressed mood is different to meeting the diagnosis criteria for depression. | I had low mood... So, I was emotionally feeling quite fragile, getting a bit teary (Participant 2). |
| Depersonalization | Depersonalization is the development of negative and callous attitudes about the people that one works with (Maslach and Jackson, 1982). | ... So, it was almost like–when we’re with our clients. We have to be [slight pause as the participant gathers thoughts] completely opened and all our stuff is way back... We are putting everything into um our clients. I think there’s a bit of resentment for that and there was almost like intrusiveness on their behalf… (Participant 4). |
| Reduced Productivity and Motivation | People measure productivity according to the rate of effective, productive effort and output. Motivation is the driving force responsible for the initiation, persistence, direction, and vigor of goal-directed behavior. Data may be coded if clinical psychologists have expressed decreased motivation to undergo any activity, and their productivity is specifically related to their work. | ... One of the main things I remember is driving to work and just wishing that I didn’t have to go just wishing I felt sick that day or, you know, that I get a flat tyre or something just so I didn’t have to go in–an excuse if you like… (Participant 2). |
| Insomnia | Insomnia is the inability to fall asleep or to maintain restful sleep. Mentions of insomnia, any previous problems or any potential problems (real or imagined) with sleeping should be coded. | … My sleep is shocking. I have to put it down to the job because when I’m on holiday, I sleep beautifully... I do really struggle with that, despite all my knowledge about sleep hygiene... my brain just won’t switch off! (Participant 1). |

## Table 3. Precursors of Burnout.

| Key Definitions  Precursors: Physical or mental conditions that precede the onset of burnout. | | |
| --- | --- | --- |
| Sub-Theme Label | **Definition/Description** | **Example Quotation/s From the Raw Data** |
| Excessive Workload and Hours of Work | Workload is the amount of work to be done, especially in a specified period. Hours of work refer to the number of hours per week that a worker performs. The term excessive is subjective and depends on clinical psychologists’ lived experiences. For example, if he/she describes a situation as being overwhelming and mentions a certain number of hours worked, then his/her workload is ‘excessive.’ | It’s like you’re a student at a university, you’ve got your final exams or something coming up, you know, in the two or three weeks beforehand you think: ‘Holy shit, I’m not ready for interviews ‘cos I haven’t done any work’... Then, by Sunday night, at least the first draft is finished ‘cos then I can put it away and my [partner], who is a language expert and qualified in editing, and my Practice Manager, reads them and pencils things in and whatever else... we have a look at the report again, and we go through the whole thing… (Participant 6).  Probably at the end of October two-thousand-and-thirteen, I was very highly aware of it [burnout]. Um–we had a big holiday planned, so I knew it was um–coming up - you know, a three-month period off… and then there was a lot of time trying to get everything prepared for that–doing a lot of contact hours (Participant 1). |
| Life Stresses | Stressors are events or experiences that result in stress. People experience ‘stress’ themselves. Life stresses should not be confused with the theme mental stress. The researcher should code datum to the sub-theme life stresses if a clinical psychologist:   - mentions an event that occurs before having burnout, - says something increased the severity of their burnout, - does not indicate when he/she experienced a particularly stressful event or experience or - describes an ambiguous stressor before having burnout.   Transference between clients’ and their psychologists is excluded from this code. | Um, and I suspect one of the things is that um, I previously used to work a lot more hours. In terms that I worked at a university full-time and then I worked private practice part-time (Participant 4).  … Because of his disability [refers to a relative], sometimes he can be a bit of a handful at times and has a sixth sense like, oh, you know like kids with behavioral problems do–If I’m stressed out, suddenly he behaves really badly. It’s like I have this extra layer that’s hard to manage…  (Participant 3). |
| Mismanagement | Mismanagement refers to poorly or wrongly managing one’s work. Most participants should be able to describe their decisions (or omissions) that result in positive or negative outcomes. The coder’s knowledge and personal experience should determine how well he/she considers participants manage each situation. | … I’m one of those people who’s had gym memberships for long periods and certainly last year I was in this habit of–I decided if I was gonna work a twelve or fourteen-hour day, I needed to go to the gym before and after… I found I often got caught up (Participant 3).  ... With electronic media that is difficult ‘cos, I get those clients who think they can call at any time of the day... with mobile phones and email access and so on–some of my clients do take full advantage of that, and that’s a bit stressful at times too (Participant 1). |
| Transference Between Clients and Clinical Psychologists | Transference involves the redirection of emotions and attitudes from the client to the psychologist. It usually has features carried over from earlier relationships. | It does um–it depends on… the context of the work–in other words, the client presenting problems, the intensity of that context in terms of its severity (Participant 6).  … I do work with quite a few very... complex cases of personality disorder–If you’ve got too many of them on your books it’s a really–um, rapid contributor… (Participant 1). |

## Table 4. Protective Factors of Burnout.

| Key Definitions  Protective Factors: Reduce the likelihood that burnout will occur. | | |
| --- | --- | --- |
| Sub-Theme Label | **Definition/Description** | **Example Quotation/s From the Raw Data** |
| Knowledge and Years Worked in Direct Care | Knowledge is anything that is known or an experience while working. Datum should be coded to this theme if those factors directly relate to the participant. | When I went to [university] you could register after two years [to become a psychologist] … of a doctorate and start working, which I did... I started working part-time while I was completing my thesis (Participant 2).  I’ve been in practice for 32 years (Participant 6).  I think that’s partly just down to experience– you know, I’ve been in this game for a while… (Participant 1). |
| Trusting Long-Term Relationships | Trust is the belief or reliance of one person on another. The term ‘long-term’ is subjective and refers to more than one year in duration. The term ‘relationship’ is used to refer to the way that people behave towards each other. | I’ve had the same therapist for like 20 years, which is kind of helpful ‘cos it makes it really easy to get to the bottom of things (Participant 3).  ... If there’s something going on for you that’s affecting your work they’ll [clinical supervisors] be able to handle it without, sort of, shaming you… (Participant 2). |

## Table 5. Barriers to Overcoming Burnout.

| Key Definitions  Barriers: Insurmountable obstacles that interfere with the satisfaction of need/s, for example, environmental or individual barriers. | | |
| --- | --- | --- |
| Sub-Theme Label | **Definition/Description** | **Example Quotation/s From the Raw Data** |
| The Fallacy of Clients’ Expectations and Needs Being More Important Than the Practicing Psychologists’. | Behavior that is driven by meeting the need/s or expectation/s of anyone other than the clinical psychologist is coded. Expectations are the beliefs that something will happen in the future. Needs are motivational states that result from a person requiring something. | Yeah, ha ha. Well, you’ve got to [do a lot to treat burnout] because otherwise you don’t–you can’t keep working in your field.... You have to be able to um meet the aim of the work, and you have to be able to–It’s unethical to not be at your best mentally when you’re working with people who are unwell (Participant 1).  So, I’m very aware of it [burnout], and I think–um feedback from colleagues and my supervisor has been that I haven’t made any poor decisions (Participant 3). |
| The Financial Cost of Working in Private Practice | Costs refer to both financial costs and opportunity costs. Financial costs are amounts that have to be paid or spent to buy or obtain something. An opportunity cost is a financial cost of something regarding an opportunity forgone. Private practice refers to the work of clinical psychologists who are self-employed. | I had to rearrange finances and pay off a few things… didn’t take on clients (Participant 4).  We’ve got all these great IT systems now…the more you outsource people to help you, the more you have to work… it’s kind of like this double-edged sword (Participant 3). |
| Contemporary Knowledge and Inadequate Education About Self-Care. | Knowledge is anything that is known. Education is the process of receiving or giving systematic instruction, especially at a school or university. Self-care refers to the provision of medical or other necessary care by the clinical psychologist. | ... We are talking about nine/ ten [thinking out-loud]– 19 years ago [more confident tone of voice]. Quite a while ago [that the participant went to University]. Yep... I know things have changed a bit since then [chuckles]... (Participant 1). |
| Time Constraints | Time constraints refer to limitations or restrictions on one’s time. | My [partner] was in the hospital in a high-risk pregnancy... she was in the hospital for two months, and then, umm our baby was born prematurely [clears throat] and then he was in a hospital for a month... Umm and what else?–Yeah, I was limited in what I could do because I would spend as much time at the hospital as I could... (Participant 5). |
